# Patient-Reported Outcome Measures and Biomechanical Variables That May Be Related to Knee Functions Following Total Knee Arthroplasty

**DOI:** 10.3390/bioengineering11090938

**Published:** 2024-09-19

**Authors:** Hannah Seymour, Fangjian Chen, Naiquan (Nigel) Zheng

**Affiliations:** Department of Mechanical Engineering and Engineering Science, University of North Carolina at Charlotte, 9201 University City Blvd., Charlotte, NC 28223, USA; hstokes3@charlotte.edu (H.S.); fchen6@charlotte.edu (F.C.)

**Keywords:** knee, data analysis, implant, daily activities, improvements

## Abstract

Total knee arthroplasty (TKA) is a commonly performed surgery aimed at alleviating pain and improving functionality. However, patients often face uncertainties in selecting the timing, location, and type of TKA implant that best meets their needs. This study aims to comprehensively compare various variables, explore trends, and identify factors potentially influencing TKA outcomes. A cohort of 40 TKA subjects received either unilateral posterior stabilized (Persona) TKA or bi-cruciate stabilized (Journey II) TKA. Additionally, 20 healthy controls matched for age, gender, and BMI were included. Participants underwent patient-reported outcome assessments, range of motion evaluations, balance assessments, proprioception tests, and biomechanical analyses. These analyses covered motion, loading, and electromyography during five daily activities and two clinical tests. Multifactor ANOVA was utilized to compare 283 variables and assess their impact on TKA outcomes. A knee biomechanics index was formulated to evaluate deviations from healthy norms. Significant differences were observed in EMG varus/valgus rotation during both ramp-up and ramp-down phases between the two implant groups. Although significant improvements were noted post-TKA for both implants, the results remained below those of the control group. Gender, age, and BMI exhibited noticeable effects on TKA outcomes across several biomechanical variables and demonstrated significant disparities compared to the controls.

## 1. Introduction

Osteoarthritis (OA) is a common joint disease that often occurs in the knee. In end-stage OA, total knee arthroplasty (TKA) can provide significant pain relief, restore mobility, and function and provide quality long-term results. TKA is a widely performed surgical procedure designed to alleviate pain and improve physical function for patients with advanced knee OA. Despite evidence that TKA generally improves functional performance, many patients continue to experience pain and dissatisfaction with their ability to perform household tasks and recreational activities [1]. A study reported that a patient’s pain level could be influenced and potentially predicted by their ability to return to full functional capacity [2]. This study aims to investigate various factors that influence the success of the surgery.

Several factors influence the choice of implant, as patients aim for the best possible outcome. A recent study has shown that subjective measures of function based on patient-reported outcome measures (PROM), directly reflect objective biomechanical measures in TKA patients [3]. While recommendations for implants include various advantages and disadvantages, the final choice often depends on the provider. Abnormal translations or rotations of the tibial component of the TKA may cause the increased possibility of implant wear or imbalance in limbs [4]. Longevity is an important factor to consider because it impacts lifespan, deterioration, and pain level. Electromyography (EMG) has been used to study patients muscle activity and prolongation time during level walking in elderly women following TKA [5]. Surgeons often have preferences for specific implants, so patients may end up with those implants by default if their doctor specializes in them. It is important to consider exploring other implant options to address an individual’s functional needs. Improving knee function and reducing pain after surgery remain primary goals for both the patient and physicians.

The emphasis of this study is comparing unilateral posterior stabilized (PS) and bi-cruciate stabilized (BCS) TKA. Patients with posterior stabilized TKA showed less posterior femoral rollback of the lateral condyle compared to normal knee but more normal tibiofemoral axial rotation and greater weight-bearing flexion [6]. The posterior cruciate ligament plays an important role in maintaining femoral rollback and internally rotating the tibia relative to the femur during flexion [7]. PS was designed to stabilize the anteroposterior knee motion by mechanical interactions between the femoral cam and the tibial post as the posterior cruciate ligament is sacrificed. BCS TKA intends to reproduce the normal knee function by implementing a two cam-post mechanism, which substitutes for both cruciate ligaments [8]. The main additional mechanical feature of the BCS is an anterior femoral cam which aims to prevent excessive posterior movement of the femur on the tibia. It is critical to determine the most suitable implant for each patient because it will lead to long term success.

Oftentimes there are so many questions that come up as a subject is trying to determine the best implant. There are often many similarities and only minor differences between implant types. Several questions are posed such as when is the best time to get surgery? Or what variables create the best chance for a successful surgery? Or is there a universal implant for everyone? Or should an implant be individualized based on age, gender, occupation, culture, and leisure activities? These questions motivate this study to examine lots of variables to see which matters most. With artificial intelligence systems to help parse through the massive amount of data from previous patients it becomes feasible for individualized treatment based on age, gender, occupation, culture, and leisure activities.

Artificial intelligence is increasingly being used in medicine to analyze medical data and uncover insights that can enhance health outcomes and patient experiences. Machine learning in healthcare can be used by medical professionals to develop better diagnostic tools to analyze medical images. Although the application of artificial intelligence in medicine is still emerging, it holds tremendous promise for advancing various fields. For TKA patients, both subjective measures (surveys and questionnaires) and objective measures (clinical and biomechanical assessment) are used to predict the most suitable implant for their specific needs. This use of personalized medicine could revolutionize the field by tailoring treatments to individual patient profiles.

This study explored the PROM and biomechanical variables and their connections of two groups of patients with PS TKA (Persona, Zimmer) and BCS TKA (Journey II, Smith & Nephew). Several studies have compared the difference between Persona and Journey II implants, finding various conclusions. A study showed that patients with BCS TKA showed more normal-like kinematics and better clinical results than those with PS TKA [9]. Other variables that were impacted because of the implant type were: functional score, tibiofemoral anterior posterior translation, axial rotation, and knee flexion, among others [10,11,12]. This study will compare PROM, clinical tests, medical records, joint biomechanics, and the EMG of five daily activities and two commonly used clinical testing activities. The comparison across implants will hopefully give guidance and help with the data collection of TKA patients, which would enable the use of artificial intelligence and precise medicine. 

The aim of this study is to investigate variables that impact the success of TKA by comparing variables between patients with Persona or Journey II implants, and the control group. The hypothesis was that patients with Persona and Journey II implants had no significant differences after 6 months post-operation. The variables that may impact the success of TKA were also investigated. Hopefully this study will lay a foundation for future collaborative data collection with more patients with all kinds of implants. A better understanding of this connection between subjects and recovery could help care providers find the right match of implant for each future patient.

## 2. Materials and Methods

This study had the goal of age and body mass index (BMI) matching 20 pairs of subjects who underwent a unilateral TKA with PS implant (Persona, Zimmer Biomet, Warsaw, IN, USA), with BCS implant (Journey II, London, UK), and a control group, respectively. The study protocol was approved by an institutional review board at the University of North Carolina at Charlotte, NC, USA; all subjects gave informed consent and received permission from their surgeons to perform testing. Participants with lower joint diseases, additional diagnosed OA of the hip or ankle, chronic pain at the contralateral knee of the TKA side, BMI greater than 38, or neurological diseases were excluded from the study. This study involved recruiting 72 subjects to form 20 age-, gender-, and BMI-matched pairs. This included 20 subjects with Journey II implants, 24 subjects with Persona implants, and 20 healthy controls. Additionally, eight subjects dropped out of the study for a variety of reasons, such as opting not to have the surgery, moving, or losing contact. After two implant groups were recruited and tested, we then recruited and tested age-, gender-, and BMI-matched controls. The TKA subjects were tested pre-operation, received an optional lab visit or phone call at 1-month post-operation, and received a 6-months post-operation and 1-year post-operation phone call. Eleven subjects came into the lab at 1-month post-operation (six Persona; six Journey II). It is important to note that all the 6-months post-operation follow-ups took place later than 6 months, with the average being 9 months, due to shutdowns caused by the COVID-19 pandemic. The study included PROM, functional tests, and motion and EMG data collection and analysis during five daily activities and two clinical testing activities. The subjects participated in many different events throughout the study (Table 1).

The study began with the subjects filling out general medical history and demographics forms. They then filled out the Knee Society Score Form and the Short Form Survey-12 scores pre-operation, 1-month post-operation, 6-monthss post-operation and 1-year post-operation. The Forgotten Joint Score Form is also filled out 1-month post-operation, 6-monthss post-operation, and 1-year post-operation. Additionally, the flexion and extension of each subject’s knee joint are measured using a goniometer in both active and passive positions, both pre-operation and post-operation.

The Biodex balance test (Biodex, Balance System SD, New York, NY, USA) involves four different procedures with each procedure having three trials. The goal of the test, regardless of the settings, is for the subject to keep the board balanced based on their center of mass. The first procedure involves balancing on a static board with both feet. The second procedure involves balancing on a dynamic board with both feet. The third and fourth procedures require the subject balancing on their left and right foot, respectively, on a static board. For the proprioception assessment, a dynamometer (Biodex Pro4, New York, NY, USA) was used to test the subject’s ability to position the knee joint at a consistent angle. The main procedure included two sections, the active angle reproduction test (active) and threshold to detect passive movement (passive). In each section, the subject performed the test at two different angles, 30 and 70 degrees, with three trials for each angle.

**Figure 1:**
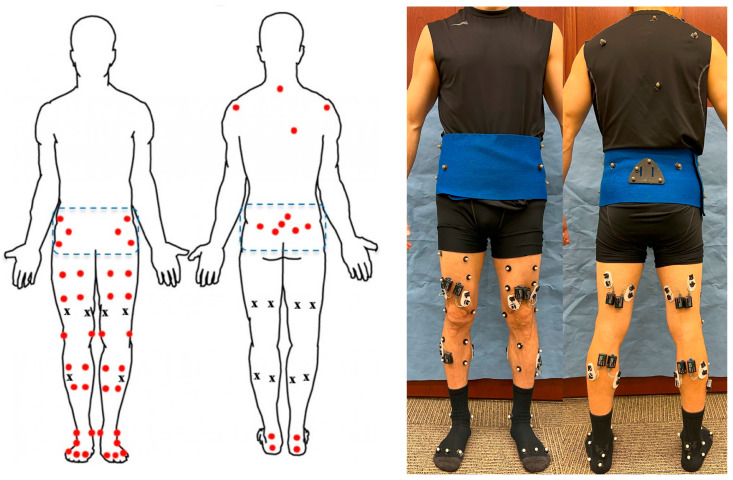
Sketch of location for motion capture markers (circles) and EMG sensors (x) on the front and back of a subject with the dotted lines indicating the fixed belt (**left**) and front and back view of a subject with markers and EMG sensors attached (**right**).

The subject participated in level walking, ramping up, ramping down, stair ascending, and stair descending, time-up-and-go (TUG) and 10-times-sit-to-stand test while their ground reaction forces, motion, and EMG data were collected at 1200 Hz, 120 Hz, and 1500 Hz, respectively. A 10-camera motion capture system (VICON, Oxford, UK), a 16-channel EMG system (Noraxon, Scottsdale, AZ, USA), and two force plates (AMTI, Watertown, MA, USA) were used to record data. For motion analysis, 52 reflective markers were attached bilaterally to the subject. There were six markers on the foot, two markers on the ankle, four markers on the shank, four markers on the thigh, two markers on the knee joint, and the same configuration on both sides. On the waist and hip, there were twelve markers, and three of them were fixed on the rigid marker set; also, there were four markers on the patients’ backs (Figure 1). For the EMG, electrodes were placed on gastrocnemius lateral and medial, vastus lateralis and medialis, biceps femoris, semitendinosus, and tibialis anterior on both legs. Subjects were instructed to perform five daily activities for five trials at a self-selected speed. They were also instructed to perform TUG test for three trials, giving their best efforts. Additionally, subjects completed the ten-times-sit-to-stand test for one trial, also giving their best efforts.

The motion trajectories, ground reaction forces, and moment data were used to calculate the joint kinematics, joint kinetics, and the EMG variables. Joint kinematics were derived from the motion of lower body segments included foot, tibia, femur, and pelvic regions with the defined local coordinates system. The joint centers of the ankles, knees, and hips were predicted as the midpoint between the lateral and medial malleoli and between the lateral and medial femoral epicondyles and markers on the pelvic region [13]. The motion of each segment during daily activities was then calculated using a least mean square-based algorithm based on the motion of the skin markers attached to that segment [14,15]. Three-dimensional joint angles (flexion/extension, varus/valgus, and internal/external rotation) were calculated using the projection method plane [16]. The three-dimensional joint moments (flexion/extension, abduction/adduction, and external/internal) were calculated using a standard inverse dynamics approach with segment mass and inertia properties obtained from empirical equations based on the subject’s weight and height. A gait cycle from both limbs of each trial was used for analysis and time normalized to 100% of the gait cycle [17]. The gait analysis variables used were the gait speed, stride length, and step length. EMG data were filtered using a high-pass zero-lag 4th-order Butterworth filter, then full-wave rectified, and finally root mean square-filtered with a smoothing window [18]. For the EMG analysis there were seven muscles on each leg analyzed using the peak, root mean square, and bilateral ratios for all activities.

A knee biomechanics index was utilized to assess knee health, based on variances in knee kinetics and kinematics variables from healthy controls. The variables incorporated into the knee biomechanics index include the following: the flexion–extension range of motion, the varus–valgus range of motion, the internal–external range of motion, the flexion–extension moment, the abduction–adduction moment, the internal–external moment, and the bilateral ratio of the knee contact force. Due to their significant impacts on performance, the flexion–extension range of motion, the flexion–extension moment, and the bilateral ratio of knee contact force were each allocated two points, while the remaining four variables were assigned one point each. 

To evaluate post-TKA surgery improvement, a subject’s data were compared to values from healthy controls. The knee biomechanics index for the healthy control group was standardized to a perfect score of 10 for each activity. An individual’s knee biomechanics index score was calculated using the healthy controls’ means and standard deviations (SDs). For variables in the sagittal plane where higher values are preferred, participants with values above the mean − SD received two points, those between mean − SD and mean − 2 SD received one point, and those below mean − 2 SD received zero points. For variables in other planes where lower values are preferred, participants with values below the mean + SD received one point, those between mean + SD and mean + 2 SD received half a point, and those above mean + 2 SD received zero points. Regarding the bilateral ratio of knee contact force, where the optimal value is one, subjects with values between mean − SD and mean + SD received two points. Scores between mean − 2 SD and mean − SD or mean + SD and mean + 2 SD were given one point, while scores below mean − 2 SD or above mean + 2 SD received zero points. 

The operative data, doctor’s notes, and medical images were also collected. The operative data include the implant size referring to the femoral component, tibial component, polyethylene insert, patellar button, and tibial stem. The doctor’s notes varied for all subjects regarding surgery and recovery. The medical images included X-rays taken both pre-operation and 6-months post-operation.

The statistics involve comparing the two implants, Persona and Journey II, pre-operation, 1-month post-operation, and 6-months post-operation for all the variables defined above using SPSS (SPSS 27; IBM). The variables compared included the patient-reported outcome measure forms, range of motion, proprioception, balance, and biomechanical testing. Further effects of BMI and age on two implants were investigated using multifactor ANOVA to compare the two implants (Persona and Journey II) post-operation using SPSS. The alpha was set to 0.05.

## 3. Results

There were 283 variables compared across Persona and Journey II and statistically significant results pre-operation and 6-months post-operation. The demographics of Persona and Journey II are quite similar. There was only a slight variation between age, gender, height, and weight (Table 2), especially in the healthy controls. This serves as a strong baseline with all subjects at the same starting place.

The analysis shows that there were many statistically significant variables pre-operation, 1-month post-operation, and 6-months post-operation. From the variables compared, 13 variables were significantly different pre-operation and 7 variables were significantly different 6-months post-operation. The significantly different variables between Persona and Journey II varied between pre-operation, 1-month post-operation, and 6-months post-operation (Table 3).

Both the Persona and Journey II self-reported forms (Knee Society Score and Short Form Survey-12) showed significant differences between the healthy control group for pre-operation and post-operation TKA surgery. However, there were no significant differences between Persona and Journey II. For the Forgotten Joint Score form there were no significant differences between Persona and Journey II for any follow-up times. Both Persona and Journey II had similar trends for all the self-reported forms.

The importance of the variables being closer to the control group shows that these subjects are close to the healthy general population. For the significant pre-operation variables, there were five variables where Persona was closer to the control group and eight variables where Journey II was closer to the control group. For the significant post-operation variables, there were four variables where Persona was closer to the control group and three variables where Journey II was closer to the control group. All other variables comparing Persona and Journey II showed some trends but did not show any significant differences.

**Figure 2:**
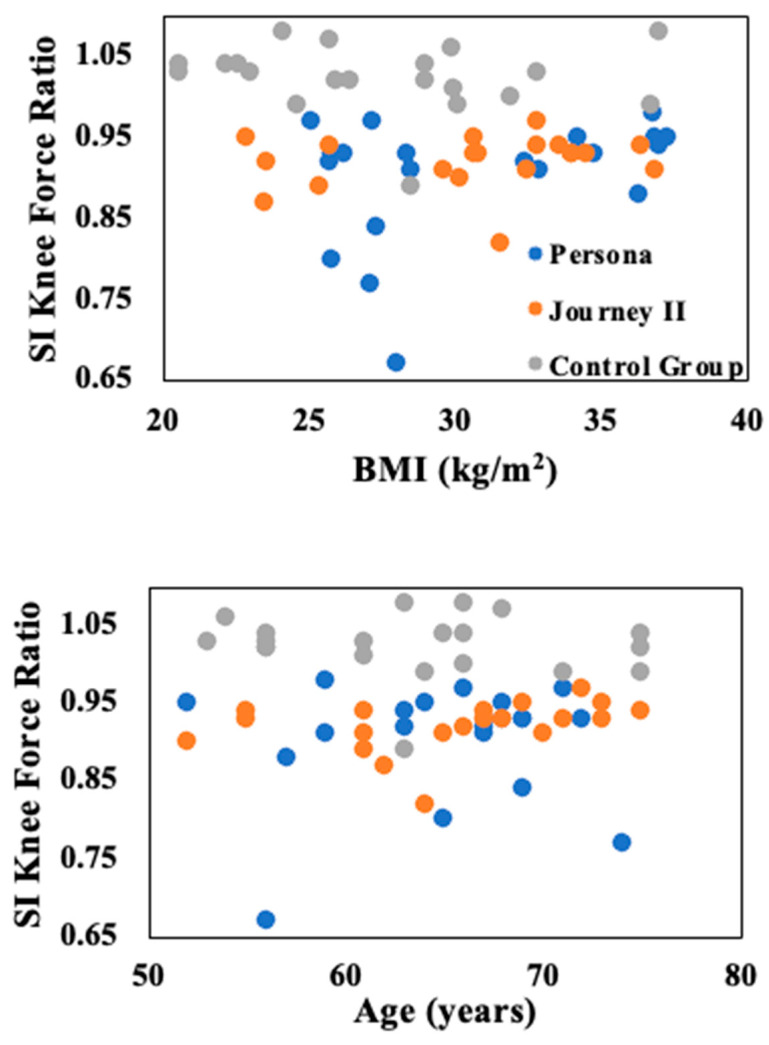
Cross plots for BMI (kg/m^2^) and age (years) for the bilateral ratio of the superior/inferior (SI) knee force during level walking.

The most obvious trend between age and BMI for the biomechanical variables was for the bilateral ratio of the superior/inferior knee force (Figure 2). The control group remained closest to 1 as that means their two legs are symmetrical. Although there was no significant difference, the Journey II group was closer to 1 than the Persona group. The trend is most obvious in level walking but continued through all five daily activities (level walking, ramp-up, ramp-down, stair ascent, and stair descent).

**Figure 3:**
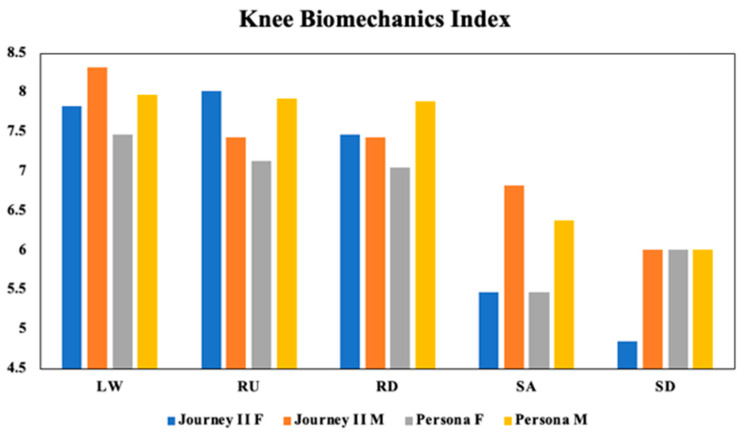
Knee biomechanics index for both implant types for female (F) and male (M) patients, with the maximum index score being 10 during level walking (LW), walking ramp-up (RU) or ramp-down (RD), and stair ascending (SA) and stair descending (SD).

The knee biomechanics index reveals variations across activities and highlights the impact of gender on TKA surgery outcomes (Figure 3). Trends indicate that gender has effects on the performance, with the most pronounced difference observed in the stair ascent. Although gender trends were evident, they did not reach statistical significance (*p* = 0.057). The type of implant also showed no significant difference on the knee biomechanics index (*p* = 0.907). However, the index performance alone dropped significantly for stair ascent and descent (*p* < 0.001).

## 4. Discussion

The primary hypothesis was rejected because there were no significant differences between Persona and Journey II at 6 months post-operation. However, several variables were identified as significantly impacting the success of the surgery. There were notable pre-operation differences in variables between the Persona and Journey II groups, likely due to slight demographic variations. The focus of this study was on post-operation variations, with significant variables including the root mean square EMG in the tibialis anterior for ramp-up, stair ascent, and stair descent, as well as a peak and valley varus/valgus rotation during both ramp-up and ramp-down walking. Identifying these significant variables is crucial to understanding what factors influence the success of TKA surgeries.

The post-operation variables with significant differences between two implants were observed during more demanding tasks, such as stair ascent and descent, rather than during simply level walking. Variations in varus/valgus rotation can have significant implications for implant wear. One study found that abnormal valgus or varus positioning of the tibial component of a TKA implant may increase the risk of loosening or implant wear, potentially leading to revision surgery [19]. In the ramp-up test, the Journey II implant group performed more similarly to the control group, whereas in the ramp-down test, the Persona implant group was closer to the control group. A previous study suggested that the kinematic differences through single fluoroscopic images could be linked to variations in the articular surface geometry [20], which may differ due to design of the two implants. While previous studies have reported that the muscle activity of quadriceps significantly affects knee performance during daily activities, none have specifically addressed variation in the tibialis anterior muscle activity [18]. This variation could be due to compensation mechanisms following surgery.

When comparing the Persona and Journey II implants, it is important to consider the variations in the implant design. Research has shown that posterior-stabilized knee implants were designed to reduce polyethylene insert wear, resulting in high surgical success rates [21]. This success rate might make posterior-stabilized implants a preferred choice, particularly for patients with an incompetent or attenuated PCL [22]. The patient’s pre-surgery health is crucial in determining the most suitable implant and likelihood of surgical success. Some differences observed between Persona and Journey II may be attributed to their distinct designs, particularly the post-cam shape of the two implants. Careful consideration is necessary when selecting the best implant design for each patient and when developing future implant designs.

Two additional questions we aimed to address pertain to BMI and age. The first question is whether a patient should lose weight before undergoing TKA surgery. While this is often recommended, the ideal BMI remains unclear. Following trends, considering the patient’s post-surgery goals, and evaluating other factors are crucial in addressing this question. Machine learning techniques could be particularly valuable in providing insights. The second question is determining the optimal timing for surgery. This is a complex issue, as it involves balancing the patient’s pain level with the longevity of the implant. Generally, patients, especially younger ones, are encouraged to delay surgery to reduce likelihood of needing a revision surgery; however, this can lead to compensatory injuries. Further investigation with a larger sample size or database could offer more clarity. The potential benefits of using artificial intelligence in this field are vast.

There is an abundance of self-reported forms, many of which are redundant. In this study, we used the Knee Society Score, the Short Form Survey-12, and the Forgotten Joint Score because they are comprehensive and widely recognized. However, artificial intelligence could radically transform this process by making it more interactive for subjects. This enhancement could lead to a more individualized approach, potentially providing a better assessment of knee joint function after TKA surgery.

The findings of this study highlight the importance of both subjective and objective measures in assessing functional improvement in TKA patients. Numerous factors influence TKA outcomes, including general health, BMI, age, knee function, and more [23]. Given the complexity of these variables, the use of artificial intelligence could be highly beneficial. Additionally, developing a knee biomechanical index could enhance TKA outcomes by predicting results, creating personalized matches, and assisting in surgical planning. While conducting a large-scale motion analysis study may not be feasible, creating a comprehensive database could help identify key markers and variables linked to the success of the surgery. Artificial intelligence could leverage patient data to predict outcomes of future TKA patients. However, before artificial intelligence can be fully integrated, it is crucial to explore all relevant variables to make more informed decisions—one of the primary goals of this study. Given the vast number of variables, traditional analysis methods can only handle a small portion of the data, limiting our ability to extract meaningful insights.

There were a few minor limitations in this study. Firstly, the COVID-19 pandemic led to an extension of the study timeline, causing variability in the timing of patient testing. Since the TKA surgery was non-essential, subject availability was affected by cancellations and postponements. Additionally, achieving perfect matching in gender, age, and BMI between the implant and control groups proved challenging, resulting in some variations. Although the groups were closely matched, slight differences in these variables pre-operation may have reflected slightly different baseline conditions. Another limitation was that we did not monitor the individual completion of rehabilitation, though all subjects completed their rehabilitation and received permits from their doctors before the post-operation lab visit. Future research should investigate the causes of differences in knee joint movement and aim to develop optimal implant designs. Longer follow-up studies on TKA patients may provide further insights into knee biomechanics during daily activities.

The Persona and Journey II implants had significant differences in several variables: root mean square EMG in the tibialis anterior for ramp-up, stair ascent, and stair descent; and peak and valley varus/valgus rotation during both the ramp-up and ramp-down tests. Artificial intelligence could be a valuable tool for precision medicine in this context. Developing a database of past subjects would enable the creation of a system to predict the most suitable implants for future TKA patients.

## Figures and Tables

**Table 1:** List of events for the TKA subjects throughout the entire study. Data collected from the control subjects were the same as those from the TKA subjects at pre-op laboratory visit, except for medical images and doctor notes. * Collected in the laboratory only if the subject came 1-month post-operation, which is optional.

Study Activity		Pre-Operation Laboratory Visit	Operation	Post-Operation		
				1-Month	>6-Months Laboratory Visit	1-Year
Demographics/Medical History		X				
Patient-Reported Outcome Measures	Knee Society Score Form	X		X	X	X
	Short Form Survey-12 Scores	X		X	X	X
	Forgotten Joint Score Form			X	X	X
Clinical Testing	Range of Motion Test	X		X *	X	
	Biodex Balance Test	X		X *	X	
	Proprioception Test	X		X *	X	
	Timed-Up-Go, 10-Times-Sit-to-Stand	X		X *	X	
Biomechanical Testing with Motion, EMG and Ground Reaction Forces during walking on level, slope and stairs		X		X *	X	
Operative Data (implant size for all components)			X			
Medical Images and Doctor Notes		X			X	

**Table 2:** Demographic variables compared between Persona and Journey II.

	Persona	Journey II	Control
Age (years)	64.9 ± 5.8	65.4 ± 6.5	63.4 ± 7.5
Gender (M/F)	11/9	11/9	11/9
BMI (kg/m^2^)	30.7 ± 4.6	30.6 ± 4.3	27.1 ± 4.4

**Table 3:** Statistically significant differences between Persona and Journey II pre-operation, 1-month post-operation, and 6-months post-operation; n is subject number with Persona/Journey II implant, where (^a^) means that Persona is closer to control group, and (^b^) means that Journey II is closer to control group.

Variables	Pre-Operation (*n* = 20/20)	>6 Months Post-Operation (*n* = 20/20)
Bilateral Ratio of Peak Muscle Activities	Ramp-Up Vastus Medialis ^b^ Ramp-Down Vastus Medialis ^b^ Semitendinosus ^b^	
Bilateral Ratio of Root Mean Square EMG	Level Walking Semitendinosus ^b^, Biceps Femoris ^b^ Ramp-Up Biceps Femoris ^b^, Tibialis Anterior ^b^	
Root Mean Square EMG	Ramp-Up Vastus Lateralis ^a^ Stair Ascent Gastrocnemius Lateral ^a^	Ramp-Up Tibialis Anterior ^a^ Stair Ascent Tibialis Anterior ^a^ Stair Descent Tibialis Anterior ^b^
Peak Rotations	Flexion/Extension Rotation Ramp-Up ^b^	Varus/Valgus Ramp-Up ^b^ Varus/Valgus Ramp-Down ^a^
Peak Forces	Medial/Lateral Force Ramp-Down ^a^ Superior/Inferior Force Ramp-Down ^a^ Medial/Lateral Force Stair Ascent ^a^	

## Data Availability

The data presented in this study are available on request from the corresponding author due to privacy or ethical restrictions.

## References

[1] Benedetti M.G., Catani F., Bilotta T.W., Marcacci M., Mariani E., Giannini S. (2003). Muscle activation pattern and gait biomechanics after total knee replacement. Clin. Biomech..

[2] Sullivan M., Tanzer M., Reardon G., Amirault D., Dunbar M., Stanish W. (2011). The role of presurgical expectancies in predicting pain and function one year following total knee arthroplasty. Pain.

[3] Biggs P.R., Whatling G.M., Wilson C., Holt C.A. (2019). Correlations between patient-perceived outcome and objectively-measured biomechanical change following Total Knee Replacement. Gait Posture.

[4] Weale A.E., Feikes J., Prothero D., O’Connor J.J., Murray D., Goodfellow J. (2002). In vitro evaluation of the resistance to dislocation of a meniscal-bearing total knee prosthesis between 30° and 90° of knee flexion. J. Arthroplast..

[5] Lee A., Park J., Lee S. (2015). Gait analysis of elderly women after total knee arthroplasty. J. Phys. Ther. Sci..

[6] Cates H.E., Komistek R.D., Mahfouz M.R., Schmidt M.A., Anderle M. (2008). In vivo comparison of knee kinematics for subjects having either a posterior stabilized or cruciate retaining high-flexion total knee arthroplasty. J. Arthroplast..

[7] Andriacchi T.P., Stanwyck T.S., Galante J.O. (1986). Knee biomechanics and total knee replacement. J. Arthroplast..

[8] Ward T.R., Burns A.W., Gillespie M.J., Scarvell J.M., Smith P.N. (2011). Bicruciate-stabilised total knee replacements produce more normal sagittal plane kinematics than posterior-stabilised designs. J. Bone Jt. Surg. Br..

[9] Inui H., Taketomi S., Yamagami R., Kono K., Kawaguchi K., Takagi K., Kage T., Tanaka S. (2020). Comparison of intraoperative kinematics and their influence on the clinical outcomes between posterior stabilized total knee arthroplasty and bi-cruciate stabilized total knee arthroplasty. Knee.

[10] Brilhault J., Ries M.D. (2010). Measuring patellar height using the lateral active flexion radiograph: Effect of total knee implant design. Knee.

[11] Murakami K., Hamai S., Okazaki K., Wang Y., Ikebe S., Higaki H., Shimoto T., Mizu-Uchi H., Akasaki Y., Nakashima Y. (2018). In vivo kinematics of gait in posterior-stabilized and bicruciate-stabilized total knee arthroplasties using image-matching techniques. Int. Orthop..

[12] Tomite T., Saito H., Kijima H., Ishikawa N., Hatakeyama Y., Tazawa H., Miyakoshi N., Shimada Y. (2021). Evaluation of anteroposterior accelerometric change after bi-cruciate stabilized total knee arthroplasty and posterior stabilized total knee arthroplasty. Knee.

[13] Yocum D.S., Valenzuela K.A., Standifird T.W., Cates H.E., Zhang S. (2022). Altered biomechanics in bilateral total knee replacement patients during stair negotiation. Knee.

[14] Spoor C., Veldpaus F. (1980). Rigid body motion calculated from spatial co-ordinates of markers. J. Biomech..

[15] Wang H., Zheng N. (2010). Knee Joint Secondary Motion Accuracy Improved by Quaternion-Based Optimizer With Bony Landmark Constraints. J. Biomech. Eng..

[16] Wang H., Fleischli J.E., Hutchinson I.D., Zheng N. (2014). Knee Moment and Shear Force Are Correlated With Femoral Tunnel Orientation After Single-Bundle Anterior Cruciate Ligament Reconstruction. Am. J. Sports Med..

[17] Gao B., Cordova M.L., Zheng N. (2012). Three-dimensional joint kinematics of ACL-deficient and ACL-reconstructed knees during stair ascent and descent. Hum. Mov. Sci..

[18] Mizner R., Snyder-Mackler L. (2005). Altered loading during walking and sit-to-stand is affected by quadriceps weakness after total knee arthroplasty. J. Orthop. Res..

[19] Werner F.W., Ayers D.C., Maletsky L.P., Rullkoetter P.J. (2005). The effect of valgus/varus malalignment on load distribution in total knee replacements. J. Biomech..

[20] Ishibashi T., Tomita T., Yamazaki T., Tsuji S., Yoshikawa H., Sugamoto K. (2021). Kinematics of bicruciate and posterior stabilized total knee arthroplasty during deep knee flexion and stair climbing. J. Orthop. Res..

[21] Papagiannis G.P.T.M., Roumpelakis I.P.T., Triantafyllou A.P.T.M., Makris I.P.T.M., Babis G.M.D.P.D. (2016). No differences identified in transverse plane biomechanics between medial pivot and rotating platform total knee implant designs. J. Arthroplast..

[22] Conditt M.A., Noble P.C., Bertolusso R., Woody J., Parsley B.S. (2004). The PCL significantly affects the functional outcome of total knee arthroplasty. J. Arthroplast..

[23] Clement N.D., Burnett R. (2013). Patient satisfaction after total knee arthroplasty is affected by their general physical well-being. Knee Surg. Sports Traumatol. Arthrosc..

